# *Dictyostelium* Differentiation-inducing Factor Derivatives Reduce the Glycosylation of PD-L1 in MDA-MB-231 Human Breast Cancer Cells

**DOI:** 10.14789/jmj.JMJ22-0039-OA

**Published:** 2023-03-13

**Authors:** AIRI HIRAYAMA, HIROTAKA ISHIGAKI, KATSUNORI TAKAHASHI, YUSUKE MIURA, HARUHISA KIKUCHI, YUZURU KUBOHARA

**Affiliations:** 1 Laboratory of Health and Life Science, Graduate School of Health and Sports Science, Juntendo University, Chiba, Japan; 1 Laboratory of Health and Life Science, Graduate School of Health and Sports Science, Juntendo University, Chiba, Japan; 2 Department of Medical Technology, Faculty of Health Science, Gunma Paz College, Gunma, Japan; 2 Department of Medical Technology, Faculty of Health Science, Gunma Paz College, Gunma, Japan; 3 Division of Natural Medicines, Faculty of Pharmacy, Keio University, Tokyo, Japan; 3 Division of Natural Medicines, Faculty of Pharmacy, Keio University, Tokyo, Japan

**Keywords:** *Dictyostelium*, differentiation-inducing factor 1 (DIF-1), triple-negative breast cancer, programmed cell death 1 (PD-1), programmed death-ligand 1 (PD-L1)

## Abstract

**Objectives:**

Triple-negative breast cancer (TNBC) is a metastatic and intractable cancer with limited treatment options. Refractory cancer cells often express the immune checkpoint molecules programmed death-ligand 1 (PD-L1) and PD-L2, which inhibit the anticancer effects of T cells. Differentiation-inducing factors, originally found in *Dictyostelium discoideum*, and their derivatives possess strong antiproliferative activity, at least in part by reducing cyclin D1 expression in various cancer cells, but their effects on PD-L1/PD-L2 have not been examined. In this study, we investigate the effects of six DIF compounds (DIFs) on the expression of PD-L1/PD-L2 and cyclin D1/D3 in MDA-MB-231 cells, a model TNBC cell line.

**Methods:**

MDA-MB-231 cells were incubated for 5 or 15 h with or without DIFs, and the mRNA expression of cyclin D1, PD-L1, and PD-L2 were assessed by quantitative polymerase chain reaction (qPCR). Whereas, MDA-MD-231 cells were incubated for 12 or 24 h with or without DIFs, and the protein expression of cyclins D1 and D3, PD-L1, and PD-L2 were assessed by Western blotting.

**Results:**

As expected, some DIFs strongly reduced cyclin D1/D3 protein expression in MDA-MB-231 cells. Contrary to our expectation, DIFs had little effect on PD-L1 mRNA expression or increased it transiently. However, some DIFs partially reduced glycosylated PD-L1 and increased non-glycosylated PD-L1 in MDA-MB-231 cells. The level of PD-L2 was very low in these cells.

**Conclusions:**

Since PD-L1 glycosylation plays an important role in preventing T cells from attacking cancer cells, such DIFs may promote T cell attack on cancer cells *in vivo*.

## Introduction

The elucidation of mechanisms underlying immunosuppression by programmed cell death protein 1 (PD-1)^1)^ and its ligand, programmed death-ligand 1 (PD-L1)^2)^, has led to great progress in the field of cancer immunotherapy. PD-L1 is an immune checkpoint inhibitor that promotes immunosuppression by binding to PD-1 on immune cells^3-7)^. Normal (non-transformed) cells in the body are not attacked by immune cells, whereas cancer (transformed) cells are recognized as “non-self” and are attacked by T cells^8, 9)^. However, cancer cells expressing PD-L1 can evade immune destruction by binding to PD-1 on the surface of T cells^6, 10-12)^. After the discovery of PD-L1, the PD-L2 ligand, which has the same action as PD-L1, was also identified^7, 13)^. Nivolumab/Opdivo^®^, an anti-PD-1 antibody approved for the treatment of various types of cancer, acts by blocking the interaction of PD-1 with PD-L1 and PD-L2, thereby releasing immunosuppression and promoting the tumor-killing effect of T cells^4, 7, 14)^. Accordingly, the application of immune checkpoint inhibitors such as anti-PD-1 and anti-PD-L1/PD-L2 and inhibitors for PD-L1/PD-L2 expression is rapidly becoming a promising cancer immunotherapy approach^4-7, 15)^.

Breast cancer is the most common cancer among women, accounting for about 25% of all new female cancers each year^16, 17)^. Triple-negative breast cancer (TNBC) is a subtype of breast cancer that does not express the estrogen receptor, progesterone receptor, or human epidermal receptor 2, with clinical features that include being highly proliferative, metastatic, heterogenous, and refractory. Due to the lack of targetable receptors, targeted hormone therapies for TNBC are not an option^18-21)^. Therefore, innovative breast cancer treatments, including novel anticancer and antimetastatic drugs, are needed. PD-L1 is expressed in 20% of TNBCs, suggesting that it may serve as a therapeutic target in this disease^22-24)^.

**Figure 1:**
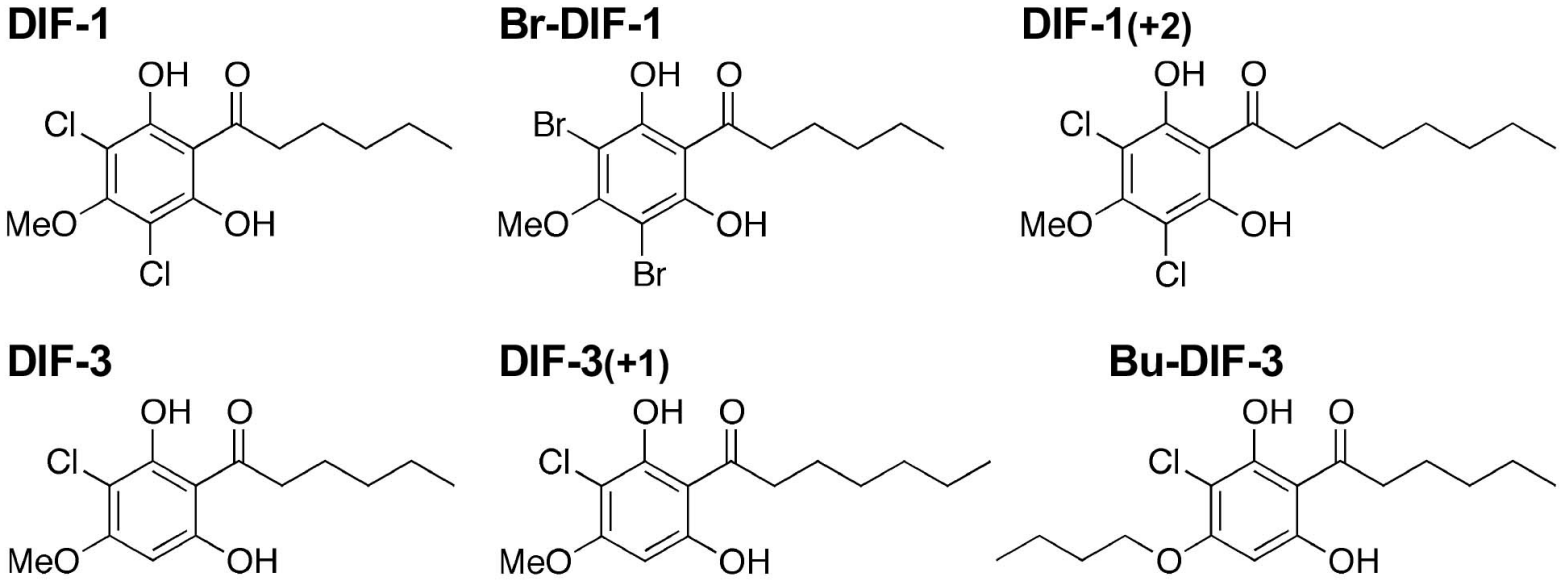
Chemical structure of six differentiation-inducing factor (DIF) compounds

Differentiation-inducing factor 1 (DIF-1, 1-(3,5- dichloro-2,6-dihydroxy-4-methoxyphenyl) hexan-1-one) and DIF-3 (1-(3-chloro-2,6-dihydroxy-4- methoxyphenyl) hexan-1-one) are chlorinated alkylphenones (Figure 1) that were originally isolated as inducers of stalk cell differentiation from the cellular slime mold *Dictyostelium discoideum*^25-27)^. Subsequently, DIFs and several of their derivatives were found to have antiproliferative and antimetastatic activities in mammalian tumor cells both *in vitro* and *in vivo*^28-42)^. Recently, we found that several DIF derivatives (of the 43 assessed) strongly suppressed the proliferation and/or serum-induced migration of MDA-MB-231 cells, a model TNBC cell line^43)^; thus, DIFs have therapeutic potential in the treatment of TNBC. Because MDA-MB-231 cells express PD-L1 and PD-L2^24, 44)^, they can be used as a model for the development of therapeutic methods and drugs targeting PD-L1/PD-L2.

In this study, we assessed the effects of six representative DIF compounds (Figure 1) on the expression of PD-L1 and PD-L2 in MDA-MB-231 cells. The results showed that although DIF compounds had little effect on PD-L1/PD-L2 protein expression or slightly decreased it, some of the DIF compounds significantly reduced glycosylated PD-L1 and increased non-glycosylated PD-L1, which may reduce PD-L1 activity. Thus, such compounds might inhibit PD-1/PD-L1 signaling, thereby facilitating activation of T cells that then attack cancer cells.

## Materials and Methods

### 1. Cells and reagents

Human MDA-MB-231 cells were obtained from American Type Culture Collection (ATCC, Manassas, VA, USA) and grown and maintained at 37°C with 5% CO_2_ and 95% air in Dulbecco's Modified Eagle Medium (DMEM) supplemented with 10% (v/v) heat-inactivated fetal bovine serum (FBS), 4.5 g/L glucose, and antibiotics (75 *μ*g/mL penicillin and 50 *μ*g/mL streptomycin). DIF compounds were synthesized as previously described^34)^ and stored as 10 mM stock solutions in dimethyl sulfoxide (DMSO) at -20°C.

### 2. Cell proliferation assay

MDA-MB-231 cells were incubated in 12-well plates (Corning, New York, NY, USA) with each well containing 1 ml of DMEM-FBS (2 × 10^4^ cells/1 mL/well) until cells attached to the bottom of the wells. After removal of the media, cells were incubated for 3 days with 1 mL DMEM-FBS containing 0.2% (v/v) DMSO or 10 or 20 *μ*M DIF compounds. Then, the cells were observed by phase-contrast microscopy and re-incubated with 1 mL of fresh DMEM-FBS containing 5% (v/v) of the cell number indicator Alamar blue (Wako Pure Chemical Industries, Osaka, Japan) until color of the media had changed. Relative cell number was determined by measuring absorbance at 570 nm (reference at 595 nm), as described previously^31, 34)^.

### 3. Quantitative polymerase chain reaction (PCR)

MDA-MB-231 cells were grown in 10 cm tissue culture dishes (Corning) to 60-80% confluence in DMEM-FBS. Then the media were removed, and cells were incubated for 5 and 15 h with 10 mL DMEM-FBS containing 0.2% (v/v) DMSO or 10 and 20 *μ*M DIF compounds. After washing the cells with 10 mL phosphate-buffered saline (PBS), cells were lysed in 1 mL Isogen (Nippon Gene Co., Ltd., Tokyo, Japan). Total RNAs were prepared from the lysates, followed by reverse transcription of 1 *μ*g RNA into cDNA by using the High-Capacity cDNA Reverse Transcription Kit (Thermo Fisher Scientific, Waltham, MA, USA) according to the manufacturer's instructions. We performed quantitative PCR (qPCR) to assess the relative mRNA levels of cyclin D1, PD-L1, and PD-L2 by using the TaqMan Gene Expression Master Mix (Applied Biosystems, Foster City, CA, USA) and LightCycler Nano Instrument (Roche, Basel, Switzerland), with glyceraldehyde-3-phosphate dehydrogenase (GAPDH) serving as the internal control. The following primers were used: cyclin D1, Hs00765553_m1; PD-L1, Hs00204257_m1; PD-L2, Hs00228839_m1; and GAPDH, Hs99999905_m1 (Thermo Fisher Scientific).

### 4. Western blot analysis

MDA-MB-231 cells were incubated in 12-well plates (Corning) with each well containing 1 ml of DMEM-FBS (5-10 × 10^4^ cells/mL/well) until cells attached to the bottom of the wells. After removal of the media, cells were incubated for 12 and 24 h with 1 mL DMEM-FBS containing 0.2% (v/v) DMSO or 10 or 20 *μ*M DIF compounds. Then cells were washed with 1 mL PBS, harvested by adding 0.1-0.2 ml of SDS-sample buffer solution (in proportion to cell density: relative cell number), crushed and heated by sonication, and used for sodium dodecyl sulfate-polyacrylamide gel electrophoresis (SDS-PAGE). Western blot analysis was performed as previously described^35)^. Briefly, proteins were resolved by SDS-PAGE and electrotransferred to membranes. After blocking with 0.3% non-fat dry-milk powder in a Tris-buffered saline (10 mM Tris-HCl, pH 7.5, 137 mM NaCl, 0.1% Tween 20; designated TBS-T hereafter) for 1 h at room temperature, the membranes were incubated for 1 h at room temperature with primary antibodies against cyclin D1, cyclin D3, PD-L1, PD-L2, or GAPDH in TBS-T. After washing with TBS-T, membranes were incubated for 1 h at room temperature with anti-mouse alkaline phosphatase or anti-rabbit IgG secondary antibody in TBS-T. Visualization of the protein bands was performed in an alkaline buffer (100 mM Tris-HCl, pH 9.5, 100 mM NaCl, 5 mM MgCl_2_) containing 5-bromo-4-chloro-3-indolyl-phosphate (62.5 *μ*g/ml) and nitro blue tetrazolium (125 *μ*g/ml); the labeled protein bands were quantified with Adobe Photoshop Element 2020 (Adobe, San Jose, CA, USA) and ImageJ software (version 1.53a).

### 5. Statistical analyses

Statistical analyses were performed using the Welch's *t*-test (two-tailed) with *p*<0.05 considered statistically significant.

## Results

### 1. Selection of six DIF compounds for evaluation

**Figure 2:**
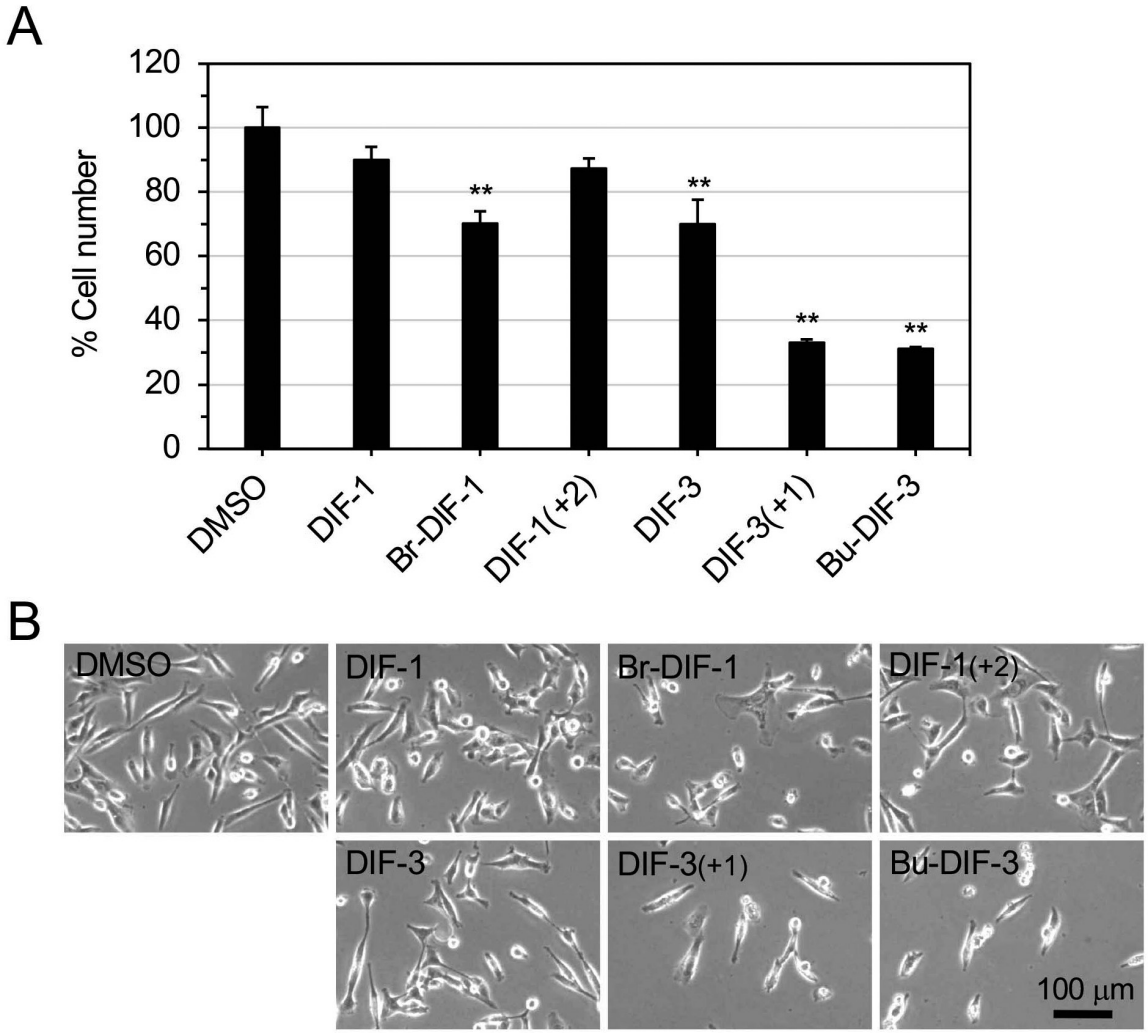
Effects of DIF compounds on cell growth and morphology in MDA-MB-231 cells. Cells were incubated for 3 days with 0.2% DMSO or 20 μM DIF-1 or its derivatives, or with 20 μM DIF-3 or DIF-3(+1) or 10 μM Bu-DIF-3, and relative cell number was determined; data are the mean ± standard deviation of a single experiment performed in triplicate (**A**). **p < 0.01 *vs.* DMSO control. Representative photos of the cells at Day 3 are shown in (**B**).

We first compared and evaluated the antitumor activity of six DIF compounds in MDA-MB-231 cells and LM8 murine osteosarcoma cells for comparison (Table 1). Because the antiproliferative and antimigratory activities of the natural compounds, DIF-1 and DIF-3, are comparatively weak in MDA-MB-231 cells and LM8 cells, we selected three compounds, one derivative of DIF-1 and two derivatives of DIF-3. Of the three compounds, Br-DIF-1 (1-(3,5-dibromo-2,6-dihydroxy-4-methoxyphenyl) hexan-1-one) is characteristic in the following points. The antiproliferative activity of Br-DIF-1 is comparatively weak in both cell lines but it strongly suppresses the serum-induced migration of MDA-MB-231 cells^43)^ and lysophosphatidic acid-induced migration of LM8 cells^36)^ (Table 1); therefore, Br-DIF-1 may be a good lead compound for the development of antimetastatic agents. Whereas, DIF-3 (+1) (1-(3-chloro-2,6-dihydroxy-4-methoxyphenyl) heptan-1-one) and Bu- DIF-3 (1-(3-chloro-2,6-dihydroxy-4-butoxyphenyl) hexan-1-one) are characteristic in the following points. The antiproliferative and antimigratory activities of DIF-3 (+1) and Bu-DIF-3 are comparatively strong in both cell lines (Table 1); therefore, DIF-3 (+1) and Bu-DIF-3 may be good lead compounds for the development of antiproliferative and antimetastatic agents. On the other hand, the antiproliferative and antimigratory activities of DIF-1 (+2) (1-(3,5-dichloro-2,6-dihydroxy-4-methoxyphenyl) octan-1-one) are weak in both cell lines (Table 1), but as this derivative has strong antimalarial activity^45)^, we used it for subsequent comparative analyses. In this study, we reconfirmed the effects of these DIF compounds on cell proliferation and morphology in MDA-MB-231 cells (Figure 2), where we used DIF-1, Br-DIF-1, DIF-1 (+2), DIF-3, and DIF-3 (+1) at 20 *μ*M and Bu-DIF-3 at 10 *μ*M since Bu-DIF-3 at more than 10 *μ*M is highly toxic to the cell. The concentrations of these compounds are omitted hereafter unless otherwise needed.

**Table 1:** Antiproliferative and antimigratory activities of DIF compounds

Compound	IC_50_ (μM) vs. Cell growth		IC_50_ (μM) vs. Cell migration	
	MDA-MB-231^43)^	LM8^36)^	MDA-MB-231^45)^	LM8^36)^
DIF-1	> 20	18.2	> 10	8.5
Br-DIF-1	> 20	18.5	3.8	5.5
DIF-1(+2)	n.d.	n.d.	> 10	n.d.
DIF-3	> 20	15.5	> 10	10.2
DIF-3(+1)	12.2	7.8	> 10	5.1
Bu-DIF-3	6.0	2.0	3.9	4.2

Footnote: n.d., not determined.

### 2. Effects of DIFs on cyclin D1 expression in MDA-MB-231 cells

**Figure 3:**
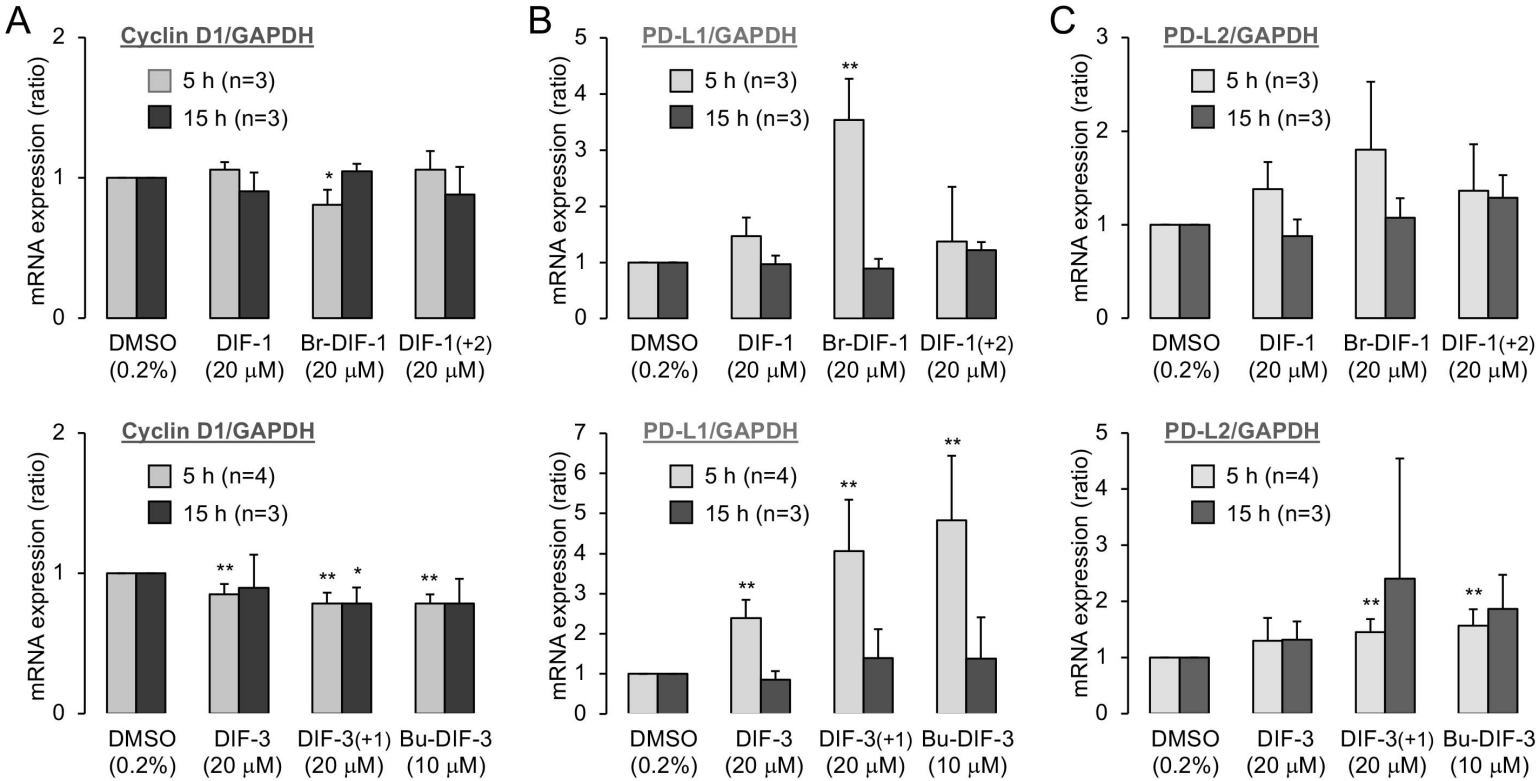
Effects of DIFs on the mRNA expression of cyclin D1 (**A**), PD-L1 (**B**), and PD-L2 (**C**) in MDA-MB-231 cells, as assessed by qPCR. Cells were incubated for 5 or 15 h in the presence of DMSO (control) or the indicated DIFs. The mean ± standard deviation of 3 or 4 independent experiments relative to control are shown. *p < 0.05, **p < 0.01 *vs.* DMSO control. GAPDH is glyceraldehyde-3-phosphate dehydrogenase.

DIF-1 and DIF-3 suppress cell proliferation, at least in part, by reducing cyclin D1 expression in many cancer cell lines^33, 35, 42, 46-48)^. To confirm the general actions of DIFs, we first evaluated the effects of the six DIF compounds on cyclin D1 mRNA expression in MDA-MB-231 cells (Figure 3A). Cyclin D1 mRNA expression was not significantly suppressed after 5 and 15 h of incubation with DIF-1 and its derivatives except for Br-DIF-1, which slightly suppressed expression at 5 h. In contrast, DIF-3, DIF-3 (+1), and Bu-DIF-3 significantly suppressed cyclin D1 mRNA expression after 5 h; after 15 h of incubation, only DIF-3 (+1) significantly suppressed expression.

**Figure 4:**
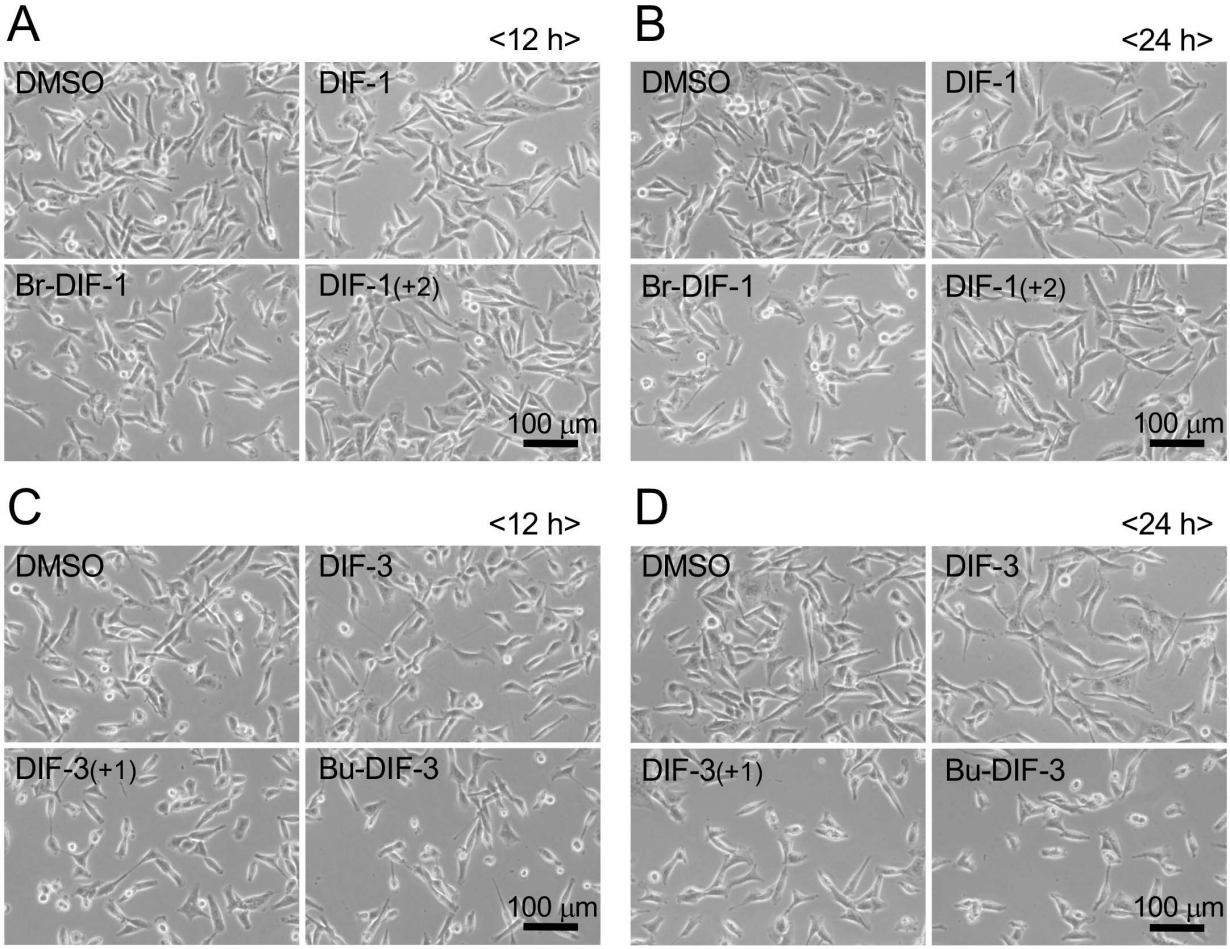
Representative phase-contrast images of MDA-MB-231 cells incubated with DIFs or DMSO control. Cells were incubated for 12 h (**A, C**) or 24 h (**B, D**) with 0.2% DMSO or 20 μM DIF-1 or its derivatives (**A, B**), or with 20 μM DIF-3 or DIF-3(+1) or 10 μM Bu-DIF-3 (**C, D**). These cells were used for Western blotting.

**Figure 5:**
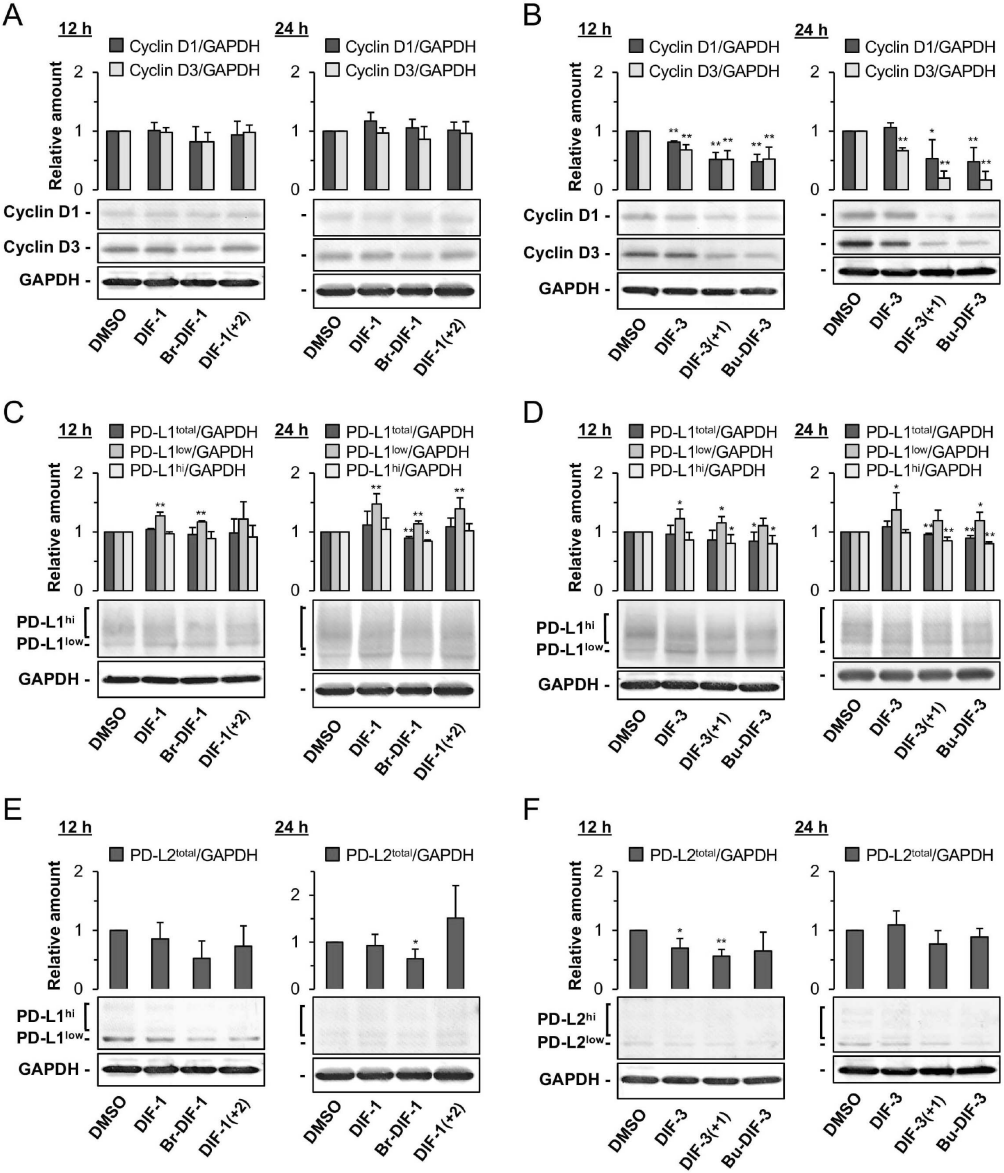
Effects of DIFs on the protein expression of cyclins D1 and D3 (**A, B**), PD-L1 (**C, D**), and PD-L2 (E, F) in MDAMB-231 cells, as assessed by Western blotting. Cells were incubated for 12 h or 24 h with 0.2% DMSO (control) or 20 μM DIF-1 or its derivatives (**A, C, E**), or with 20 μM DIF-3 or DIF-3(+1) or 10 μM Bu-DIF-3 (**B, D, F**). The amounts of cyclins D1 and D3 (A, B), the amounts of total PD-L1 (PD-L1^total^), non-glycosylated PD-L1 (PD-L1^low^), and glycosylated PD-L1 (PD-L1^hi^) (**C, D**), and the amounts of total PD-L2 (**E, F**) were normalized with GAPDH and shown relative to those with DMSO. The mean ± standard deviation of three independent experiments relative to control are shown together with representative images of the blots. *p < 0.05, **p < 0.01 *vs.* DMSO control.

Next, we examined the effects of DIFs on the protein expression of D-type cyclins D1 and D3 in MDA-MB-231 cells; note that the cells were alive and healthy up to 24 h of incubation with the DIFs (Figure 4) until the cells were collected for Western blot analysis. Expression of cyclins D1 and D3 was not significantly changed after 12 and 24 h of incubation with DIF-1 and its derivatives (Figure 5A); however, DIF-3, DIF-3 (+1), and Bu-DIF-3 significantly suppressed expression of both cyclins D1 and D3 protein after 12 and 24 h of incubation except for cyclin D1 with DIF-3 at 24 h (Figure 5B). These results showed that at the indicated concentrations, DIF-1 compounds had little effect on cyclins D1 and D3 protein expression but DIF-3 compounds reduced their expression in MDA-MB-231 cells.

### 3. Effects of DIFs on the expression of PD-L1 and PD-L2 in MDA-MB-231 cells

To verify whether DIFs might be useful for cancer immunotherapy, we assessed the effects of the six DIFs on the expression of PD-L1 and PD-L2 mRNA in MDA-MB-231 cells (Figure 3B, C). Contrary to our expectation, Br-DIF-1, DIF-3, DIF-3 (+1), and Bu-DIF-3 transiently increased PD-L1 mRNA expression after 5 h of incubation but did not significantly affect expression at 15 h (Figure 3B), whereas DIF-1 and DIF-1 (+2) did not significantly affect PD-L1 mRNA expression after 5 or 15 h of incubation. PD-L2 mRNA expression was not significantly affected after 5 and 15 h of incubation with DIF-1, its derivatives, and DIF-3, but it was slightly increased after 5 h of incubation with DIF-3 (+1) or Bu-DIF-3 (Figure 3C).

Next, we evaluated the effects of DIFs on the expression of PD-L1 (Figure 5C, D) and PD-L2 proteins (Figure 5E, F) in MDA-MB-231 cells. Because PD-L1 has four asparagine residues modified with sugar chains^49, 50)^, Western blotting detects multiple bands with different molecular weights. We quantified the amounts of total PD-L1 (PD- L1^total^), unmodified PD-L1 (PD-L1^low^, ~33 kDa), and glycosylated PD-L1 (PD-L1^hi^, max ~50 kDa). The expression of total PD-L1 protein was not significantly affected after 12 and 24 h of incubation with DIF-1, DIF-1 (+2), or DIF-3, whereas Br- DIF-1, DIF-3 (+1), and Bu-DIF-3 significantly reduced PD-L1^total^ by an average of 5-10% by 24 h (Figure 5C, D). Interestingly, however, all six DIFs tended to increase PD-L1^low^ and reduce PD-L1^hi^ at both timepoints. In particular, by 24 h Br-DIF-1, DIF-3 (+1), and Bu-DIF-3 significantly increased PD-L1^low^ by an average of 15-20% and significantly reduced PD-L1^hi^ by an average of 15-20% (Figure 5C, D), suggesting that these DIF derivatives might increase PD-L1^low^, possibly by reducing either PD-L1^hi^ or PD-L1^total^, or both. Because the protein expression of PD-L2 was low overall, only the total amount of PD-L2 protein was quantified (Figure 5E, F). Only Br-DIF-1 at 24 h and DIF-3 and DIF-3 (+1) at 12 h significantly reduced PD-L2 expression.

## Discussion

### 1. Antiproliferative and antimigratory effects of DIF on MDA-MB-231 cells

DIF-1, DIF-3, and their derivatives have antiproliferative and/or antimigratory activities in a variety of tumor cells *in vitro* and *in vivo*^28-42)^, and are expected to be lead compounds for the development of anticancer agents. We previously showed that several DIF derivatives, such as DIF-3 (+1) and Bu-DIF-3, strongly suppressed both the proliferation and serum-induced migration of MDA-MB-231^43)^ (Table 1), while another group showed that 30 *μ*M DIF-1 significantly suppressed the proliferation of MCF-7 human breast cancer cells *in vitro*, at least in part via the reduction of cyclin D1 expression^51)^. More recently, the same group has shown that 30 *μ*M DIF-1 inhibited the proliferation and migration of MDA-MB-231 cells *in vitro*, and 150 mg/kg DIF-1 (administered every 12 h for 14 days to mice) inhibited the growth and metastasis of TNBC cells *in vivo*^48)^. Herein, we confirmed that DIF-3 (+1) and Bu-DIF-3, the most promising DIF derivatives as anticancer agents evaluated in this study, may suppress cell proliferation in MDA-MB-231 cells, at least in part by reducing the expression of cyclins D1 and D3 (Figure 3A, 5B).

### 2. Suppression of PD-L1 glycosylation by DIF

As described in the Introduction section, because MDA-MB-231 cells express the immune checkpoint molecules PD-L1 and PD-L2^24, 44)^, these cells can be used for screening candidate agents targeting PD-L1/PD-L2. Here, we evaluated the effects of six DIFs (Figure 1) on the expression of PD-L1 and PD-L2 in MDA-MB-231cells (Figure 3B, C, 5C-F). Initially, we expected the DIFs to suppress expression of PD-L1 and PD-L2; contrary to our expectations, Br-DIF-1, DIF-3, DIF-3 (+1), and Bu-DIF-3 transiently increased the mRNA expression of PD-L1 at 5 h (Figure 3B), whereas the total protein expression of PD-L1 was not significantly increased after 12 and 24 h of incubation with the DIFs (Figure 5C, D). The reason for the transient increase in PD-L1 mRNA expression is unknown, but, for example, DIFs might induce a transient increase in PD-L1 mRNA expression as a secondary effect of arresting cell cycle. In any case, since protein expression is generally regulated by the balance between mRNA translation and protein degradation, any PD-L1 protein synthesized at 5 h may have been degraded to some extent by 12 h during incubation with DIFs.

**Figure 6:**
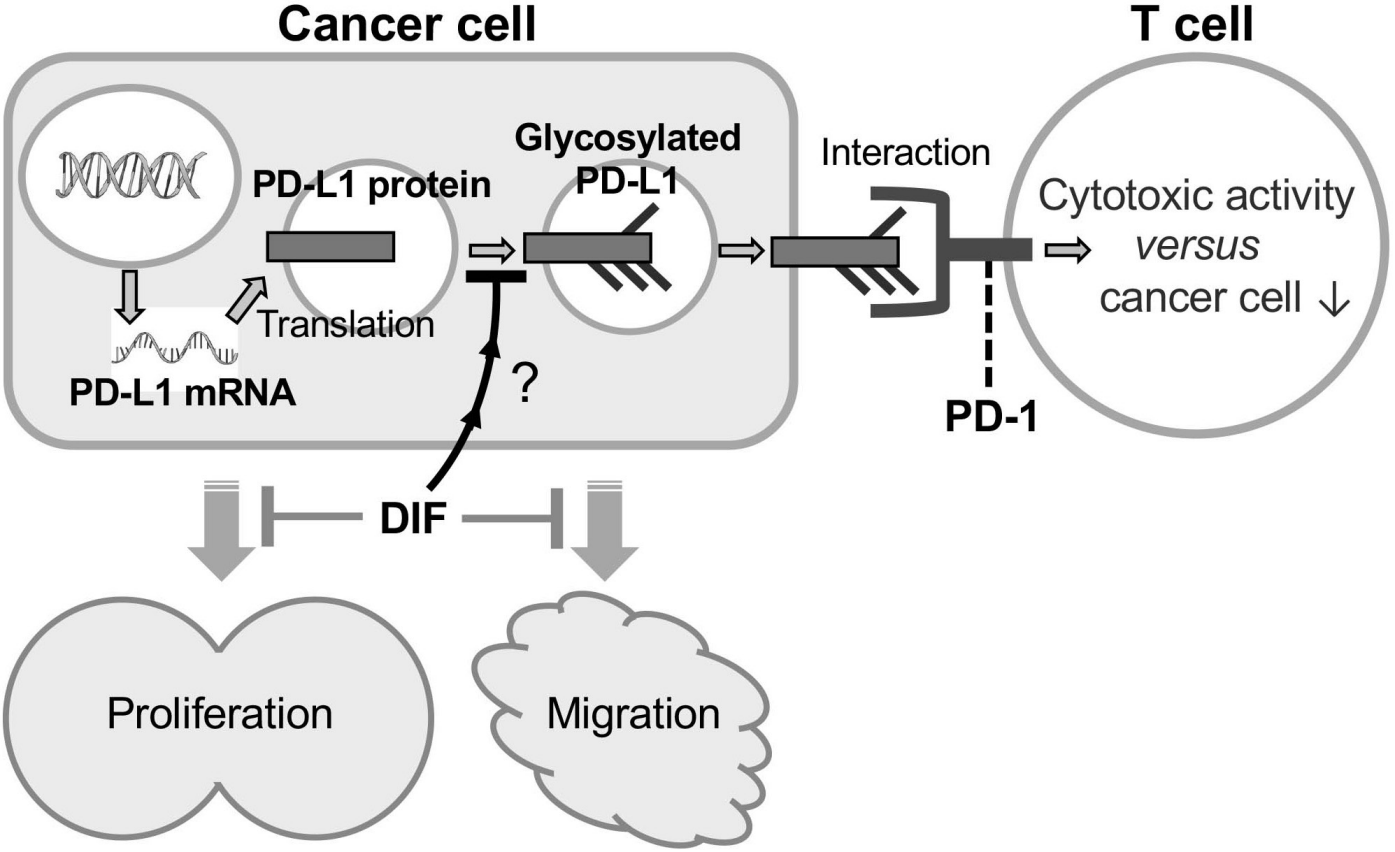
Schema of the processing pathway of PD-L1 and the action of DIFs. After translation from PD-L1 mRNA, PD-L1 protein is glycosylated at four asparagine residues (N35, N192, N200, and N219)^50)^. N-glycosylated PD-L1 binds to its receptor PD-1 on T cells, which suppresses the cytotoxic activity of T cells, thereby allowing cancer cells to escape attack from T cells. DIF might be able to weaken PD-L1/PD-1 interaction by inhibiting glycosylation of PD-L1 and thus facilitating restoration of T cell immunity to some extent. In addition, DIF can suppress cell proliferation and migration (metastasis) of some cancer cells including TNBC^36), 38), 41), 43), 48), 51)^; *e.g.* Bu-DIF-3 strongly suppresses cell proliferation and migration of MDA-MB-231 and LM8 cells (Table 1)^36), 43)^.

Interestingly, Br-DIF-1, DIF-3 (+1), and Bu- DIF-3 reduced PD-L1^total^ at 24 h (Figure 5C, D) and tended to increase the amount of PD-L1^low^, possibly by reducing PD-L1^hi^ (glycosylated PD-L1) (Figure 5C, D). Glycosylation of PD-L1 is essential for the PD-L1/PD-1 interaction and immunosuppression in TNBC^52)^. Glycosylated PD-L1 becomes resistant to degradation by the proteasome and stabilizes^49)^. Therefore, inhibition of PD-L1 glycosylation by DIFs might lead to inhibition of PD-L1/PD-1 signaling, thereby reducing immunosuppression of T cells and facilitating their attack on cancer cells (Figure 6).

The expression of PD-L2 protein was decreased by treatment with Br-DIF-1 at 24 h and DIF-3 and DIF-3 (+1) at 12 h (Figure 5E, F). However, as the expression level of PD-L2 is considerably low in MDA-MB-231 cells (Figure 5E, F), it is unknown whether these levels of PD-L2 are involved in immunosuppression of T cells.

Herein, we assessed the effects of DIF-1 (+2) on the expression of cyclins D1 and D3 and PD-L1/PD-L2 in MDA-MB-231 cells, as DIF-1 (+2) has antimalarial activity^45)^. The only significant biological activity of DIF-1 (+2) in these cells was a significant increase in PD-L1^low^ (Figure 5C).

## Conclusions

In this study, we assessed the effects of six DIF compounds on the expression of PD-L1 and PD-L2 in MDA-MB-231 cells and found that Br-DIF-1, DIF-3 (+1), and Bu-DIF-3 reduced PD-L1 glycosylation. Our results suggest that such compounds might inhibit PD-L1/PD-1 signaling and thereby reduce immunosuppression of T cells, thus facilitating their attack of cancer cells (Figure 6), although it is currently unknown whether such a DIF-induced reduction in glycosylation actually affects T cell activity. Accordingly, DIFs may be good lead compounds for the development not only of antiproliferative and antimigratory (antimetastatic) drugs but also of immunotherapy drugs against TNBC and possibly some other metastatic cancers. It may be worth investigating the inhibitory effects of other DIF derivatives (~50 DIF derivatives in total)^34, 40, 43)^ on PD-L1 and PD-L2 protein expression in MDA-MB-231 cells as well as in other cancer cells.

## Funding

This work was supported in part by JSPS KAKENHI Grant (No. 19K07139 to YK) and the Joint Research Program of Juntendo University, Faculty of Health and Sports Science (to YK).

## Author contributions

Conceptualization, YK; methodology, HK and YK; validation, AH and YK; data analysis and investigation, AH, HI, KT, YM and YK; synthesis of compounds, HK; original draft preparation, KT and YK; review and editing, AH, HI, YM and HK. All authors have read and approved the final manuscript.

## Conflicts of interest statement

The authors declare no conflict of interest.

## References

[1] Ishida Y, Agata Y, Shibahara K, Honjo T: Induced expression of PD-1, a novel member of the immunoglobulin gene superfamily, upon programmed cell death. EMBO J, 1992; 11: 3887-3895. 10.1002/j.1460-2075.1992.tb05481.x PMC556898 1396582

[2] Freeman GJ, Long AJ, Iwai Y, et al: Engagement of the PD-1 immunoinhibitory receptor by a novel B7 family member leads to negative regulation of lymphocyte activation. J Exp Med, 2000; 192: 1027-1034. 10.1084/jem.192.7.1027 PMC2193311 11015443

[3] Nishimura H, Minato N, Nakano T, Honjo T: Immunological studies on PD-1 deficient mice: implication of PD-1 as a negative regulator for B cell responses. Int Immunol, 1998; 10: 1563-1572. 10.1093/intimm/10.10.1563 9796923

[4] Okazaki T, Honjo T: PD-1 and PD-1 ligands: from discovery to clinical application. Int Immunol, 2007; 19: 813-824. 10.1093/intimm/dxm057 17606980

[5] Okazaki T, Chikuma S, Iwai Y, Fagarasan S, Honjo T: A rheostat for immune responses: the unique properties of PD-1 and their advantages for clinical application. Nat Immunol, 2013; 14: 1212-1218. 10.1038/ni.2762 24240160

[6] Iwai Y, Hamanishi J, Chamoto K, Honjo T: Cancer immunotherapies targeting the PD-1 signaling pathway. J Biomed Sci, 2017; 24: 26. 10.1186/s12929-017-0329-9 PMC5381059 28376884

[7] Jubel JM, Barbati ZR, Burger C, Wirtz DC, Schildberg FA: The Role of PD-1 in Acute and Chronic Infection. Front Immunol, 2020; 11: 487. 10.3389/fimmu.2020.00487 PMC7105608 32265932

[8] Riley JL: PD-1 signaling in primary T cells. Immunol Rev, 2009; 229: 114-125. 10.1111/j.1600-065X.2009.00767.x PMC3424066 19426218

[9] Chakravarti N, Prieto VG: Predictive factors of activity of anti-programmed death-1/programmed death ligand-1 drugs: immunohistochemistry analysis. Transl Lung Cancer Res, 2015; 4: 743-7451. 10.3978/j.issn.2218-6751.2015.12.10 PMC4700222 26798583

[10] Iwai Y, Ishida M, Tanaka Y, Okazaki T, Honjo T: Minato, N. Involvement of PD-L1 on tumor cells in the escape from host immune system and tumor immunotherapy by PD-L1 blockade. Proc Natl Acad Sci USA, 2002; 99: 12293-12297. 10.1073/pnas.192461099 PMC129438 12218188

[11] Zou W, Chen L: Inhibitory B7-family molecules in the tumour microenvironment. Nat Rev Immunol, 2008; 8: 467-477. 10.1038/nri2326 18500231

[12] Schumacher TN, Schreiber RD: Neoantigens in cancer immunotherapy. Science, 2015; 348: 69-74. 10.1126/science.aaa4971 25838375

[13] Latchman Y, Wood CR, Chernova T, et al: PD-L2 is a second ligand for PD-1 and inhibits T cell activation. Nat Immunol, 2001; 2: 261-268. 10.1038/85330 11224527

[14] Topalian SL, Drake CG, Pardoll DM: Immune checkpoint blockade: a common denominator approach to cancer therapy. Cancer Cell, 2015; 27: 450-461. 10.1016/j.ccell.2015.03.001 PMC4400238 25858804

[15] Schmid P, Adams S, Rugo HS, et al: Atezolizumab and nab-paclitaxel in advanced triple-negative breast cancer. N Engl J Med, 2018; 379: 2108-2121. 10.1056/NEJMoa1809615 30345906

[16] Ferlay J, Soerjomataram I, Dikshit R, et al: Cancer incidence and mortality worldwide: sources, methods and major patterns in GLOBOCAN 2012. Int J Cancer, 2015; 136: E359-386. 10.1002/ijc.29210 25220842

[17] Hwang S-Y, Park S, Kwon Y: Recent therapeutic trends and promising targets in triple negative breast cancer. Pharmacol Ther, 2019; 199: 30-57. 10.1016/j.pharmthera.2019.02.006 30825473

[18] Perou CM, Sørlie T, Eisen MB, et al: Molecular portraits of human breast tumours. Nature, 2000; 406: 747-752. 10.1038/35021093 10963602

[19] Dent R, Trudeau M, Pritchard KI, et al: Triple-negative breast cancer clinical features and patterns of recurrence. Clin Cancer Res, 2007; 13: 4429-4434. 10.1158/1078-0432.CCR-06-3045 17671126

[20] Lehmann BD, Bauer JA, Chen X, et al: Identification of human triple-negative breast cancer subtypes and preclinical models for selection of targeted therapies. J Clin Invest, 2011; 121: 2750-2767. 10.1172/JCI45014 PMC3127435 21633166

[21] Jiao Q, Wu A, Shao G, et al: The latest progress in research on triple negative breast cancer (TNBC): risk factors, possible therapeutic targets and prognostic markers. J Thorac Dis, 2014; 6: 1329-1335. 10.3978/j.issn.2072-1439.2014.08.13 PMC4178098 25276378

[22] Sabatier R, Finetti P, Mamessier E, et al: Prognostic and predictive value of PDL1 expression in breast cancer. Oncotarget, 2015; 6: 5449-5464. 10.18632/oncotarget.3216 PMC4467160 25669979

[23] Huang X, Xie X, Wang H, et al: PDL1 and LDHA act as ceRNAs in triple negative breast cancer by regulating miR-34a. J Exp Clin Cancer Res, 2017; 36: 129. 10.1186/s13046-017-0593-2 PMC5602941 28915924

[24] Salama EA, Adbeltawab RE, El Tayebi HM: XIST and TSIX: Novel cancer immune biomarkers in PD-L1-overexpressing breast cancer patients. Front Oncol, 2020; 9: 1459. 10.3389/fonc.2019.01459 PMC6966712 31998636

[25] Morris HR, Taylor GW, Masento MS, Jermyn KA, Kay RR: Chemical structure of the morphogen differentiation inducing factor from *Dictyostelium discoideum*. Nature, 1987; 328: 811-814. 10.1038/328811a0 3627228

[26] Morris HR, Masento MS, Taylor GW, Jermyn KA, Kay RR: Structure elucidation of two differentiation inducing factors (DIF-2 and DIF-3) from the cellular slime mould *Dictyostelium discoideum*. Biochem J, 1988; 249: 903-906. 10.1042/bj2490903 PMC1148792 3355503

[27] Kay RR, Flatman P, Thompson CRL: DIF signalling and cell fate. Semin Cell Dev Biol, 1999; 10: 577-585. 10.1006/scdb.1999.0341 10706822

[28] Asahi K, Sakurai A, Takahashi N, Kubohara Y, Okamoto K, Tanaka Y: DIF-1, morphogen of *Dictyostelium discoideum*, induces the erythroid differentiation in murine and human leukemia cells. Biochem Biophys Res Commun, 1995; 208: 1036-1039. 10.1006/bbrc.1995.1438 7702602

[29] Kubohara Y, Saito Y, Tatemoto K: Differentiation-inducing factor of *D. discoideum* raises intracellular calcium concentration and suppresses cell growth in rat pancreatic AR42J cells. FEBS Lett, 1995; 359: 119-122. 10.1016/0014-5793(95)00022-2 7867781

[30] Kubohara Y: DIF-1, putative morphogen of *D. discoideum*, suppresses cell growth and promotes retinoic acid-induced cell differentiation in HL-60. Biochem Biophys Res Commun, 1997; 236: 418-422. 10.1006/bbrc.1997.6964 9240452

[31] Kubohara Y: Effects of differentiation-inducing factors (DIFs) of *Dictyostelium discoideum* on the human leukemia K562 cells: DIF-3 is the most potent anti-leukemic agent. Eur J Pharmacol, 1999; 381: 57-62. 10.1016/s0014-2999(99)00548-8 10528134

[32] Kanai M, Konda Y, Nakajima T, et al: Differentiation-inducing factor-1 (DIF-1) inhibits STAT3 activity involved in gastric cancer cell proliferation via MEK-ERK dependent pathway. Oncogene, 2003; 22: 548-554. 10.1038/sj.onc.1206109 12555068

[33] Takahashi-Yanaga F, Taba Y, Miwa Y, et al: *Dictyostelium* differentiation-inducing factor-3 activates glycogen synthase kinase-3β and degrades cyclin D1 in mammalian cells. J Biol Chem, 2003; 278: 9663-9670. 10.1074/jbc.M205768200 12522140

[34] Gokan N, Kikuchi H, Nakamura K, Oshima Y, Hosaka K, Kubohara Y: Structural requirements of *Dictyostelium* differentiation-inducing factors for their stalk-cell-inducing activity in *Dictyostelium* cells and anti-proliferative activity in K562 human leukemic cells. Biochem Pharmacol, 2005; 70: 676-685. 10.1016/j.bcp.2005.06.002 16023080

[35] Kubohara Y, Kikuchi H, Matsuo Y, Oshima Y, Homma Y: Mitochondria are the target organelle of differentiation-inducing factor-3, an anti-tumor agent isolated from *Dictyostelium discoideum*. PLoS One, 2013; 8: e72118. 10.1371/journal.pone.0072118 PMC3744471 23977224

[36] Kubohara Y, Komachi M, Homma Y, Kikuchi H, Oshima Y: Derivatives of *Dictyostelium* differentiation-inducing factors inhibit lysophosphatidic acid-stimulated migration of murine osteosarcoma LM8 cells. Biochem Biophys Res Commun, 2015; 463: 800-805. 10.1016/j.bbrc.2015.06.016 26056940

[37] Kubokura N, Takahashi-Yanaga F, Arioka M, et al: Differentiation-inducing factor-3 inhibits intestinal tumor growth *in vitro* and *in vivo*. J Pharmacol Sci, 2015; 127: 446-455. 10.1016/j.jphs.2015.03.005 25913757

[38] Oladimeji P, Kubohara Y, Kikuchi H, et al: A derivative of differentiation-inducing factor-3 inhibits PAK1 activity and breast cancer cell proliferation. Int J Cancer Clinic Res, 2015; 2: 1-6. 10.23937/2378-3419/2/4/1023 PMC4682050 26688830

[39] Arioka M, Takahashi-Yanaga F, Kubo M, Igawa K, Tomooka K, Sasaguri T: Anti-tumor effects of differentiation-inducing factor-1 in malignant melanoma: GSK-3-mediated inhibition of cell proliferation and GSK-3-independent suppression of cell migration and invasion. Biochem Pharmacol, 2017; 138: 31-48. 10.1016/j.bcp.2017.05.004 28501501

[40] Takahashi K, Kikuchi H, Nguyen VH, et al: Biological activities of novel derivatives of differentiation-inducing factor-3 from *Dictyostelium discoideum*. Biol Pharm Bull, 2017; 40: 1941-1947. 10.1248/bpb.b17-00484 29093342

[41] Kubohara Y, Kikuchi H, Oshima Y: Derivatives of *Dictyostelium* differentiation-inducing factors inhibit serum-dependent cell migration of murine osteosarcoma LM8 cells. Juntendo Med J, 2019; 65: 71-76. 10.1016/j.bbrc.2015.06.016 26056940

[42] Kubohara Y, Kikuchi H: *Dictyostelium*: an important source of structural and functional diversity in drug discovery. Cells, 2018; 8: 6. 10.3390/cells8010006 PMC6356392 30583484

[43] Totsuka K, Makioka Y, Iizumi K, et al: Halogen-substituted derivatives of *Dictyostelium* differentiation-inducing factor-1 suppress serum-induced cell migration of human breast cancer MDA-MB-231 cells *in vitro*. Biomolecules, 2019; 9: 256. 10.3390/biom9070256 PMC6681295 31261818

[44] Xu Y, Wu Y, Zhang S, et al: A Tumor-Specific Super-Enhancer Drives Immune Evasion by Guiding Synchronous Expression of PD-L1 and PD-L2. Cell Rep. 2019, 29, 3435-3447. 10.1016/j.celrep.2019.10.093 31825827

[45] Mita T, Hirai M, Maki Y, et al: Derivatives of *Dictyostelium* differentiation-inducing factors suppress the growth of *Plasmodium* parasites *in vitro* and *in vivo*. Biochem Pharmacol, 2021; 194: 114834. 10.1016/j.bcp.2021.114834 34774530

[46] Takahashi-Yanaga F, Mori J, Matsuzaki E, et al: Involvement of GSK-3beta and DYRK1B in differentiation-inducing factor-3-induced phosphorylation of cyclin D1 in HeLa cells. J Biol Chem, 2006; 281: 38489-38497. 10.1074/jbc.M605205200 17046823

[47] Akaishi E, Narita T, Kawai S, et al: Differentiation-inducing factor-1-induced growth arrest of K562 leukemia cells involves the reduction of ERK1/2 activity. Eur J Pharmacol, 2004; 485: 21-29. 10.1016/j.ejphar.2003.11.041 14757120

[48] Seto-Tetsuo F, Arioka M, Miura K, et al: DIF-1 inhibits growth and metastasis of triple-negative breast cancer through AMPK-mediated inhibition of the mTORC1-S6K signaling pathway. Oncogene, 2021; 40: 5579-5589. 10.1038/s41388-021-01958-4 34304250

[49] Li CW, Lim SO, Xia W, et al: Glycosylation and stabilization of programmed death ligand-1 suppresses T-cell activity. Nat Commun, 2016; 7: 12632. 10.1038/ncomms12632 PMC5013604 27572267

[50] Wang YN, Lee HH, Hsu JL, Yu D, Hung MC: The impact of PD-L1 N-linked glycosylation on cancer therapy and clinical diagnosis. J Biomed Sci, 2020; 27: 77. 10.1186/s12929-020-00670-x PMC7333976 32620165

[51] Tetsuo F, Arioka M, Miura K, et al: Differentiation-inducing factor-1 suppresses cyclin D1-induced cell proliferation of MCF-7 breast cancer cells by inhibiting S6K-mediated signal transducer and activator of transcription 3 synthesis. Cancer Sci, 2019; 110: 3761-3772. 10.1111/cas.14204 PMC6890445 31553107

[52] Li CW, Lim SO, Chung EM, et al: Eradication of triple negative breast cancer cells by targeting glycosylated PD-L1. Cancer Cell, 2018; 33: 187-201. 10.1016/j.ccell.2018.01.009 PMC5824730 29438695

